# Potentially fatal complications of new systemic anticancer therapies: pearls and pitfalls in their initial management

**DOI:** 10.2478/raon-2024-0027

**Published:** 2024-04-14

**Authors:** Milena Blaz Kovac, Bostjan Seruga

**Affiliations:** Ljubljana Community Health Centre, Ljubljana, Slovenia; Faculty of Medicine, University of Ljubljana, Ljubljana, Slovenia; Division of Medical Oncology, Institute of Oncology Ljubljana, Ljubljana, Slovenia

**Keywords:** potentially fatal toxicity, immune checkpoint inhibitor, chimeric antigen receptor T-cells, Bispecific T-cell engager, antibody dug conjugate, immunosuppressive therapy

## Abstract

**Background:**

Various types of immunotherapy (i.e. immune checkpoint inhibitors [ICIs], chimeric antigen receptor [CAR] T-cells and bispecific T-cell engagers [BiTEs]) and antibody drug conjugates (ADCs) have been used increasingly to treat solid cancers, lymphomas and leukaemias. Patients with serious complications of these therapies can be presented to physicians of different specialties. In this narrative review we discuss potentially fatal complications of new systemic anticancer therapies and some practical considerations for their diagnosis and initial treatment.

**Results:**

Clinical presentation of toxicities of new anticancer therapies may be unpredictable and nonspecific. They can mimic other more common medical conditions such as infection or stroke. If not recognized and properly treated these toxicities can progress rapidly into life-threatening conditions. ICIs can cause immune-related inflammatory disorders of various organ systems (e.g. pneumonitis or colitis), and a cytokine release syndrome (CRS) and immune effector cell-associated neurotoxicity syndrome (ICANS) may develop after treatment with CAR T-cells or BiTEs. The cornerstones of management of these hyper-inflammatory disorders are supportive care and systemic immunosuppressive therapy. The latter should start as soon as symptoms are mild-moderate. Similarly, some severe toxicities of ADCs also require immunosuppressive therapy. A multidisciplinary team including an oncologist/haematologist and a corresponding organ-site specialist (e.g. gastroenterologist in the case of colitis) should be involved in the diagnosis and treatment of these toxicities.

**Conclusions:**

Health professionals should be aware of potential serious complications of new systemic anticancer therapies. Early diagnosis and treatment with adequate supportive care and immunosuppressive therapy are crucial for the optimal outcome of patients with these complications.

## Introduction

The outcome of patients with cancer has improved substantially over the last few decades. Most modern cancer care is delivered in the outpatient setting. Patients with cancer can develop various oncologic emergencies which can be cancer or treatment related. Patients undergoing anticancer treatment who develop acute illnesses often seek medical attention with general practitioners (GPs) and in emergency departments.^1,2^ Prompt identification of oncologic emergencies, timely intervention and coordinated follow-up with oncology care teams are crucial for optimal outcome.^3^ However, there may be a lack of knowledge about management of toxic complications of new anticancer therapies such as immunotherapy among nononcologist health providers, including emergency physicians (EPs).^4^

The most common and well-known classic oncologic emergencies are: (i) metabolic (e.g. tumour lysis syndrome [TLS], hypercalcemia, syndrome of inappropriate antidiuretic hormone), (ii) hematologic (e.g. febrile neutropenia, hyperviscosity syndrome), (iii) structural (e.g. superior vena cava syndrome, malignant epidural spinal cord compression, malignant pericardial effusion, or (iv) treatment related (e.g. chemotherapy-induced oral mucositis, radiation pneumonitis).^5^ New systemic anticancer therapies can cause life-threatening complications which may be generally less-known than classical oncologic emergencies.

In this narrative review we discuss potentially fatal complications of new systemic anticancer therapies that differ from classic oncologic emergencies and provide some practical considerations for their diagnosis and initial management. For this purpose, a comprehensive search of the literature was performed through PubMed using the following key words: “immune checkpoint inhibitor”, “CAR T-cells”, “bispecific T-cell engager”, “antibody drug conjugate”, “toxicity” and “adverse event”. Articles describing diagnosis and management of treatment-related toxicities, including recommendations of oncologic societies and working groups were included.

## Immune checkpoint inhibitors

**FIGURE 1.**
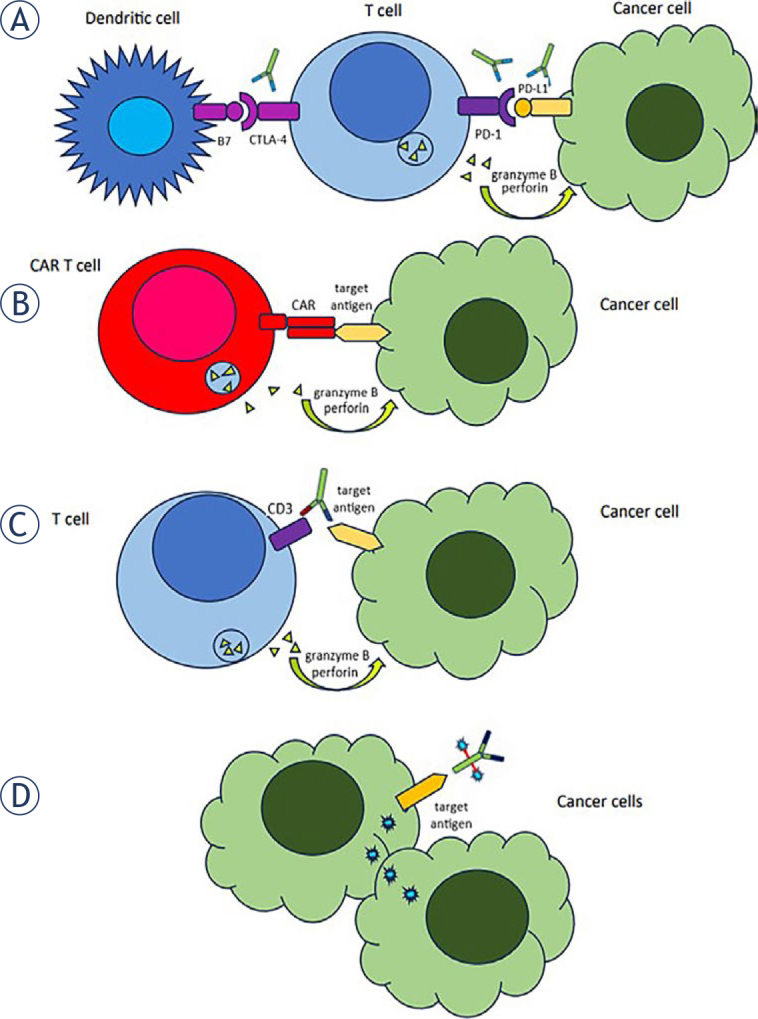
Mechanisms of action of new anticancer therapies. **(A)** Immune checkpoint inhibitor; **(B)** CAR T-cell; **(C)** Bispecific T-cell engager and **(D)** Antibody drug conjugate.

The immune checkpoint inhibitors (ICIs) are prescribed as monotherapy or in combination with chemotherapy and/or targeted anticancer agents in patients with both early and advanced solid cancers and Hodgkin’s lymphoma (Table 1). These agents are monoclonal antibodies which enhance the immune response to cancer cells. They block negative regulators of T-cell activation, such as cytotoxic T lymphocyte associated antigen 4 (CTLA-4), programmed cell death 1 (PD-1) and programmed cell death 1 ligand (PD-1L), and rein-vigorate pre-existing T-cells (Figure 1). They target neoantigens presented by the major histocompatibility complex (MHC) molecules on the surface of cancer cells. Efficacy of ICIs may be restricted due to the lack of neoantigens presented on cancer cells, defects in expression of the MHC or in other components of the antigen-presenting machinery in cancer cells, development of resistant tumour subclones and lack of T-cells in the immunosuppressive microenvironment of the tumour.^6^ ICIs are usually administered repeatedly every few weeks in the outpatient setting.

**TABLE 1.** Approved immune checkpoint inhibitors in the European Union

**Immune checkpoint inhibitor**	**Target**	**Approved indications**	

		**Early cancer**	**Advanced cancer**
Atezolizumab (Tecentriq)	PD-L1	NSCLC	Urothelial carcinoma, NSCLC, SCLC, TNBC, HCC
Avelumab (Bavencio)	PD-L1	_	Urothelial carcinoma, RCC, Merkel cell carcinoma
Cemiplimab (Libtayo)	PD-1	_	Cutaneous SCC, Basal cell carcinoma, NSCLC, Cervical carcinoma
Durvalumab (Imfinzi)	PD-L1	_	NSCLC, SCLC, HCC, Biliary tract cancer
Ipilimumab (Yervoy)	CTLA-4	_	Melanoma, RCC, NSCLC, MPM, CRC, Oesophageal carcinoma
Nivolumab (Opdivo)	PD-1	Urothelial carcinoma, melanoma, NSCLC, oesophageal and GEJ cancer	Melanoma, NSCLC, RCC, cHL, Head and neck SCC, MPM, Urothelial carcinoma, CRC, Oesophageal SCC, Gastric, GEJ or Oesophageal adenocarcinoma
Pembrolizumab (Keytruda)	PD-1	RCC, melanoma, NSCLC, TNBC	RCC, Melanoma, NSCLC, HL, Urothelial carcinoma, Head and neck SCC, Cancers with MSI-H or MMRd, Oesophageal carcinoma, Endometrial carcinoma, Cervical carcinoma, Gastric and GEJ adenocarcinoma

CRC = colorectal cancer; cHL = classical Hodgkin lymphoma; CTLA-4 = cytotoxic T-lymphocyte antigen; GEJ = gastro-oesophageal junction; HCC = hepatocellular carcinoma; HL = Hodgkin lymphoma; MMRd = mismatch repair deficiency; MPM = malignant pleural mesothelioma; NSCLC = non-small cell lung cancer; MSI-H: microsatellite instability – high; OSCC = oesophageal squamous cell carcinoma; PD-1 = program death 1; PD-L1 = program death ligand; RCC = renal cell carcinoma, SCC = squamous cell carcinoma; SCLS = small cell lung cancer; TNBC = triple-negative breast cancer

**FIGURE 2.**
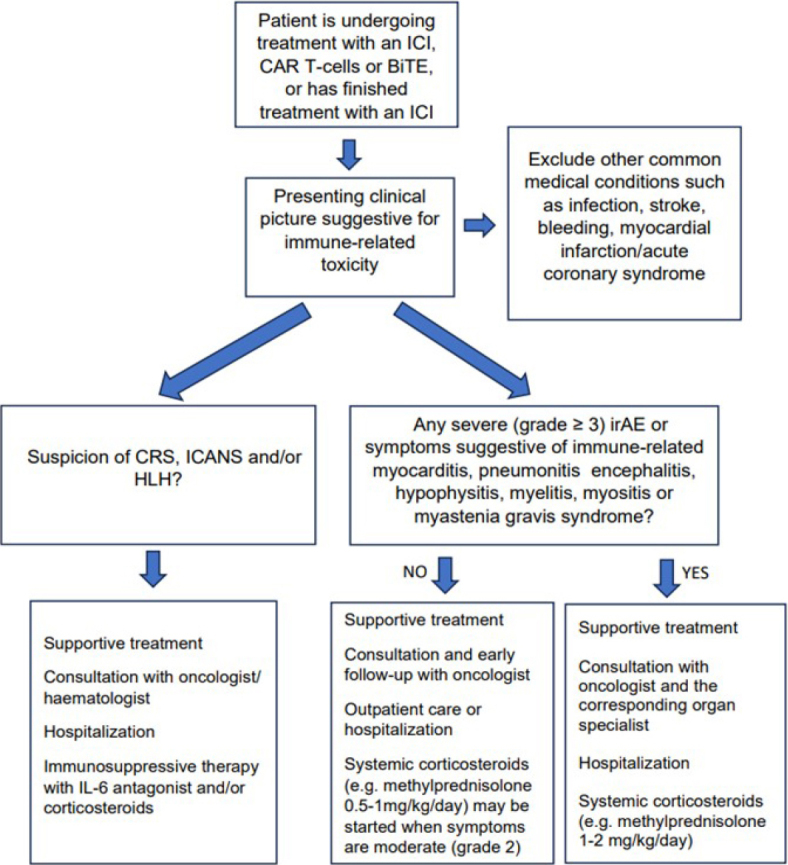
Management of toxicities of immunotherapy. BiTE = bispecific T-cell engager; CAR = chimeric antigen receptor; CRS = cytokine release syndrome; HLH = haemophagocytic lymphohistiocytosis; ICANS = immune effector-cell associated neurotoxicity syndrome; ICI = immune checkpoint inhibitor; irAE = immune-related adverse events

The ICIs have a unique toxicity profile distinct from that of chemotherapy and other targeted agents. As these agents enhance immune response they can cause immune-related adverse events (irAEs) (i.e. immune-related inflammatory disorders), which can be life-threatening. In contrast to chemotherapy and some other targeted drugs development of irAEs is more unpredictable. They can affect any organ system and can occur at any time during a patient’s treatment or sometimes long after therapy with an ICI has been discontinued.^7^ A majority of irAEs occur during the first four months of treatment with an ICI and they most commonly affect the skin, endocrine, gastrointestinal and pulmonary systems. Some other irAEs such as ICI-related myocarditis, hypophysitis, encephalitis and myositis are very rare but important cause of morbidity and mortality.^8,9^ Early identification and treatment of irAEs is crucial to limiting their severity and duration. However, the presentation of irAEs is often non-specific and can mimic other common medical conditions such as infections, stroke, intracranial bleeding and myocardical infarction. Before treatment with an irAE is started it is very important to rule out these conditions. Importantly, mild irAEs can rapidly progress to be life-threatening conditions. Therefore, when an irAE is suspected the patient’s symptoms and vital signs should be closely monitored. Detailed recommendations outlining the diagnosis, treatment and follow-up of patients with irAEs have been published by oncologic societies.^10^ In general, in patients with mild or moderate symptomatic irAEs (i.e. grade ≤ 2) symptomatic treatment in the outpatient setting is recommended and early follow-up with a treating oncologist should be arranged. Exceptions are patients with symptoms suggestive of immune-related myocarditis, neurological irAEs involving the central nervous system (i.e. hypophysitis, meningitis, encephalitis and myelitis), dyspnoea or myasthenic syndromes, who should be hospitalised immediately and treated by a multidisciplinary team involving the oncologist and the corresponding organ-site specialist (Figure 2). In patients with moderate symptoms (i.e. grade 2) systemic corticosteroids (e.g. methylprednisolone 0.5−1 mg/kg/day) are recommended. All patients with severe and life-threatening irAEs (i.e. grade ≥ 3) should be immediately hospitalized and presented to the multidisciplinary team (Figure 2).^10^ The cornerstones of management of severe irAEs are supportive care and immunosuppressive therapy with systemic corticosteroids (e.g. methylprednisolone 1−2 mg/kg/day), including initial high-dose pulse corticosteroids (e.g. methylprednisolone 500−1000 mg/day for three days) in some conditions. In some severe cases refractory to corticosteroids blocking of tumour necrosis factor (TNF)-α with infliximab, blocking of the interleukin-6 receptor (IL-6R) with tocilizumab, intravenous immunoglobulins (IVIGs) and mycophenolate mofetil may be beneficial.^9,10^ When symptoms of the irAR are severe initiation of corticosteroids cannot be postponed and empirical antimicrobial therapy can be started concurrently with corticosteroids and discontinued when infection is excluded.^10^

## Chimeric antigen receptor T-cells and bispecific T-cell engagers

CAR T-cells and BiTEs are both T-cell engaging therapies and have a similar toxicity profile. Chimeric antigen receptor (CAR) T-cells are a cell-based therapy in which patient’s T-cells are extracted by leukapheresis and then genetically modified through the insertion of DNA encoding recombinant protein CAR on their surface, expanded and then administered back to the patient. Whereas the extracellular domain of the CAR recognizes a cancer-specific antigen, its intracellular domain activates the T-cell immunogenic antineoplastic response (Figure 1).^11,12^ For example, tisagenlecleucel binds to the Cluster of Differentiation (CD)19 on B-cell leukaemia and lymphoma cells and activates the immune system to destroy malignant cells. CAR T-cells are now an established treatment for patients with relapsed and/or refractory B-cell lymphomas, B-cell acute lymphoblastic leukaemia and multiple myeloma (MM) (Table 2). CAR T-cells can engraft long-term and provide long-term ongoing responses against cancer cells. For most patients, CAR T-cell therapy is a one-time treatment. Before infusion of CAR T-cells patients receive lymphodepleting chemotherapy. Research into CAR T-cells is also extending to other diseases, including solid tumors, infections and autoimmune disorders. On average, patients are hospitalized for 12 days after infusion of the CAR T-cells.^13^ While natural antibodies have two targeting arms that bind to the same target antigen, bispecific antibodies are engineered hybrid molecules with two distinct binding domains that target two distinct antigens. Bispecific T-cell engagers (BiTEs) bind simultaneously to a selected antigen on cancer cells and to the invariant component of the T-cell receptor complex, a CD3 chain with signalling capacity (Figure 1).^14^ For example, one arm of glofitamab binds to the CD3 on T-cells and another arm to the CD20 on B-cell lymphoma cells (Table 2). BiTEs are approved for use in patients with relapsed and/or refractory B-cell lymphomas, MM and uveal melanoma (Table 2). Similar to CAR T-cells, targeting of cancer cells with BiTEs is independent of the MHC. T-cell engagement is dependent on repeated administration of the BiTEs. Premedication with systemic corticosteroids and step-up dosing reduces the risk of severe immune-related toxicities with BiTEs that are described below. It is recommended that within a step-up phase of treatment patients are hospitalized or at least remain within the proximity of a healthcare facility for a short time after the administration of a BiTE.^14,15^

**TABLE 2.** Approved CAR T cell therapies and bispecific T cell engagers in the European Union

**Agent**	**Type of therapy**	**Target**	**Indications**
Tisagenlecleucel (Kymriah)	CAR T	CD19	B-cell acute lymphoblastic leukaemia, Diffuse large B-cell lymphoma, Follicular lymphoma
Axicabtagene ciloleucel (Yescarta)	CAR T	CD19	Primary mediastinal large B-cell lymphoma, Diffuse large B-cell lymphoma, High grade B-cell lymphoma, Follicular lymphoma
Brexucabtagene autoleucel (Tecartus)	CAR T	CD19	Mantle-cell lymphoma, B-cell acute lymphoblastic leukaemia
Lisocabtagene maraleucel (Breyanzi)	CAR T	CD19	Follicular lymphoma grade 3B, Primary mediastinal large B-cell lymphoma, Diffuse large B-cell lymphoma
Idecabtagene vicleucel (Abecma)	CAR T	BCMA	Multiple myeloma
Ciltacabtagene autoleucel (Carvykti)	CAR T	BCMA	Multiple myeloma
Talquetamab (Talvey)	BiTE	GPRC5D/CD3	Multiple myeloma
Teclistamab (Tecvayli)	BiTE	BCMA/CD3	Multiple myeloma
Glofitamab (Columvi)	BiTE	CD20/CD3	Diffuse large B-cell lymphoma
Mosunetuzumab (Lunsumio)	BiTE	CD20/CD3	Follicular lymphoma
Tebentafusp (Kimmtrak)	BiTE	Gp100/CD3	Uveal melanoma
Teclistamab (Tecvayli)	BiTE	BCMA/CD3	Multiple myeloma

BCMA = B-cell maturation antigen; BiTE = bispecific T cell engager; CAR T = chimeric antigen receptor T-cells; CD3 = cluster of differentiation 3; CD19 = cluster of differentiation 19; CD20 = cluster of differentiation 20; GP100 = G protein 100; GPRC5D = G protein-coupled receptor, class C, group 5, member D

**TABLE 3.** Grades 2 and 3 of the selected immune-related adverse events (irAEs)

**irAE**	**Grade 2**	**Grade 3**
Maculo-papular rash	Papules and/or pustules covering 10−30% BSA, which may or may not be associated with symptoms of pruritus or tenderness; associated with psychosocial impact; limiting instrumental ADL; papules and/or pustules covering > 30% BSA with or without mild symptoms	Papules and/or pustules covering > 30% BSA with moderate or severe symptoms; limiting self-care ADL; associated with local superinfection with oral antibiotics indicated
Diarrhoea/enterocolitis	Increase of 4−6 stools/day over baseline	Increase of ≥ 7 stools/day over baseline
ILD/Pneumonitis	Symptomatic (presence of new or worsening symptoms: dyspnoea, cough), medical intervention indicated, limiting instrumental ADL	Severe symptoms, oxygen indicated, limiting self-care ADL
Rheumatologic toxicity	Moderate pain, stiffness and/or weakness limiting instrumental ADL	Severe pain, stiffness and/or weakness limiting self-care ADL
Neuro-muscular toxicity	Moderate pain associated with weakness, limiting instrumental ADL	Pain associated with severe weakness, limiting self-care ADL
Hepatotoxicity	ALT or AST 3−5 × ULN	ALT > 5 × or AST < 20 × ULN
Renal toxicity	Serum creatinine >1.5−3 × above the baseline or the UNL, KDIGO stage 2: increase in serum creatinine 2−2.9 × above the baseline	Serum creatinine > 3 × above the baseline or > 3−6 × ULN, KDIGO stage 3: increase in serum creatinine > 3 × or to > 4.0 mg/dl or initiation of dialysis

ALT = alanine transaminase; ADL = activities of daily living; AST = aspartate aminotransferase; BSA = body surface area; ILD = interstitial lung disease; KDIGO = kidney disease improving global outcomes; ULN = upper limit normal

The cytokine release syndrome (CRS) or cytokine storm is a result of activated T-cells, other immune cells and vascular endothelial cells, which results in the overproduction of inflammatory cytokines. It typically manifests in the first week after therapy, rarely later.^16^ While some patients experience mild, flu-like symptoms others may experience more severe and potentially life-threating complications similar to septic shock and multi-organ failure. CRS usually presents with a fever which may not respond to antipyretics, and hypotension, headache, hypoxia, rash and organ dysfunction.^17^ In contrast to CRS, immune cell-associated neurologic syndrome (ICANS) is less frequent than CRS. It usually manifests around seven days after administration of therapy, rarely several weeks later, and it is often associated with preceding CRS. Patients with ICANS may present with an altered level of consciousness, aphasia, impairment of cognitive skills, motor weakness, seizures, and cerebral oedema.^17,18^ The pathophysiology of ICANS is associated with the accumulation of pro-inflammatory cytokines and CAR T-cells in the central nervous system, together with the activation of resident glial cells.^19^ The T-cell engaging therapies are not only associated with CRS and ICANS, but can also cause various hematologic toxicities, including haemophagocytic lymphohistiocytosis (HLH), prolonged myelosuppression, coagulopathies and tumor lysis syndrome (TLS).^20^ Presentation of HLH may be similar to CRS and is characterized by a fever, cytopenia, splenomegaly, jaundice, and the pathologic finding of haemophagocytosis in bone marrow and other tissues. CRS usually precedes HLH by a few days but in rare cases HLH can develop weeks after resolution of the CRS.^21^ When toxicity of T-cell engagers is suspected a treating haematologist/oncologist should be consulted and the patient should be admitted to the hospital, preferably to the cancer centre. Mild CRS and ICANS are often self-limited with proper supportive care but can rapidly progress into life-threatening conditions, which may require management in the intensive care unit (ICU). Patients with these complications require close vigilance and prompt pharmacological treatment when there is no adequate response to supportive care and/or in the case of moderate or severe symptoms (grade ≥ 2) (Table 4).^18,19,20,21^ Supportive care includes antipyretics, intravenous hydration and symptomatic management of organ toxicities and constitutional symptoms in patients with CRS and intravenous hydration and aspiration precautions in patients with ICANS. When there is a combination of fever and hypotension, which does not require vasopressors (i.e. grade 2) CRS should be managed with the IL-6R antagonist tocilizumab (Figure 2). Although there is limited experience with additional therapies, alternate IL-6R antagonists such siltuximab and clazakizumab or the IL-1 receptor antagonist anakinra may be used for CRS refractory to tocilizumab.^22–23^ Systemic corticosteroids should be added only in refractory, prolonged, or higher-grade CRS. Patients with moderate symptoms of ICANS and/or mild somnolence awakening to voice (i.e. grade 2) should be treated with systemic corticosteroids (e.g. dexamethasone 10 mg bid). In severe cases initial high-dose pulse corticosteroids (e.g. methylprednisolone/day 500−1000 mg of for three days) are recommended. When ICANS and CRS occur concurrently tocilizumab should be used with caution as it can lead to deterioration of ICANS (Figure 2).^22^ The cornerstones of treatment of HLH are corticosteroids and an IL-6R antagonist. Both of these are contraindicated in patients with severe infection and underline the importance of arriving at a clear diagnosis prior to the initiation of treatment. In severe cases of HLH additional therapy with etoposide should be considered.^24^ Prolonged cytopenias can be treated with growth factor support and corticosteroids, and infections due to prolonged B-cell aplasia with infusion of IVIGs. Management of disseminated intravascular coagulation (DIC) is supportive, in the case of severe DIC corticosteroids and an IL-6R antagonist can be used.^22^

**TABLE 4.** Grades of the cytokine release syndrome (CRS) and the immune cell-associated neurologic syndrome ICANS

**Toxicity**	**Grade 1**	**Grade 2**	**Grade 3**	**Grade 4**
CRS	Fever: ≥ 38°C Hypotension: none Hypoxia: none	Fever: ≥ 38°C AND Hypotension: not requiring vasopressor AND/OR Hypoxia	Fever: ≥ 38°C AND Hypotension: requiring vasopressor AND/OR Hypoxia	Fever: ≥ 38°C AND Hypotension requiring multiple vasopressors AND/OR Hypoxia requiring positive pressure
ICANS	ICE score: 7−9 No depressed level of consciousness	ICE score: 3−6 AND/OR Mild somnolence awaking to voice	ICE score: 0−2 AND/OR Depressed level of consciousness awakening only to tactile stimulus AND/OR clinical seizure focal or generalized that resolve with intervention AND/OR Focal or local oedema on neuroimaging	ICE sore: 0 AND/OR Stupor or coma AND/OR Life-threatening prolonged seizure AND/OR Diffuse cerebral oedema on neuroimaging, decerebrate or decorticate posturing or papilledema, cranial nerve VI palsy, or Cushing’s triad

Immune effector cell-associated encephalopathy (ICE) assessment tool: (A) Orientation: orientation to year, month, city, and hospital: 4 points. (B) Naming: ability to name three objects: 3 points. (C) Following commands: ability to follow simple commands: 1 point. (D) Writing: ability to write a standard sentence: 1 point. (E) Attention: ability to count backward from 100 by 10: 1 point

## Antibody-drug conjugates

Antibody-drug conjugates (ADCs) have been described as ‘magic bullets’ of cancer treatment. The rationale behind the design of ADCs is to achieve targeted delivery of cytotoxic molecules by linking them to antibodies targeting tumour-specific antigens with the expectation of less toxicity than conventional chemotherapy (Figure 1). For example, enfortumab vedotin is a nectin-4-directed antibody and microtubule inhibitor monomethyl auristatin E conjugate. The use of antibody-drug conjugates (ADCs) is expanding rapidly, with development moving progressively from lymphomas and leukaemias to various solid cancers, and from monotherapy to combination strategies (Table 5).^25,26^

**TABLE 5.** Approved antibody drug conjugates in the European union and their potentially fatal toxicities

**Antibody drug conjugate**	**Target/cytotoxic agent**	**Indication**	**Potentially fatal complications**
Belantamab mafodotin (Blenrep)	BCMA/mcMMAF	Multiple myeloma	Pneumonitis Thrombocytopenic bleeding
Brentuximab vedotin (Adcetris)	CD30/MMAE	Hodgkin and non-Hodgkin lymphoma	Progressive multifocal encephalopathy (reactivation of JCV) Pancreatitis ILD/Pneumonitis/ARDS Serious infections/Opportunistic infections Severe skin reactions (SJS, TEN) Liver failure Tumor lysis syndrome
Gemtuzumab ozogamicin (Mylotarg)	CD33/ ozogamicin	AML	Liver failure (VOD/SOS) Myelosuppression Tumour lysis syndrome
Inotuzumab ozogamicin (Besponsa)	CD22/ ozogamicin	B-cell ALL	Liver failure (VOD/SOS) Myelosuppression Tumor lysis syndrome
Loncastuximab tesirine (Zynlonta)	CD19/ PBD	DLCBCL	Opportunistic infections Oedema and effusions
Polatuzumab vedotin (Polivy)	CD79b/ MMAE	DLCBCL	Neutropenic infection Opportunistic infection Progressive multifocal encephalopathy Tumor lysis syndrome
Enfortumab vedotin (Padcev)	Nectin-4/ MMAE	Advanced urothelial carcinoma	Severe skin reactions (SJS, TEN) ILD/Pneumonitis Hyperglycaemia/Diabetic ketoacidosis
Trastuzumab deruxtecan (Enhertu)	HER-2/ Dxd	Advanced breast, non-small cell lung and gastric cancer	Pneumonitis/ILD Neutropenic infection
Trastuzumab emtansine (Kadcyla)	HER-2/ Emtansine	Early and advanced breast cancer	Liver failure Haemorrhagic events ILD/Pneumonitis
Sacituzumab govitecan (Trodelvy)	Trop-2/ SN-38	Advanced breast cancer	Neutropenic infection Severe diarrhoea

ALL = acute lymphoblastic leukaemia; AML = acute myeloid leukaemia; ARDS = adult respiratory distress syndrome; BCMA = B-cell maturation antigen; CD = cluster of differentiation; DLCBCL = diffuse large cell B-cell lymphoma; Dxd = an exatecan derivative and a topoisomerase I inhibitor; HER-2 = human epidermal growth factor receptor 2; ILD = interstitial lung disease; JCV = John Cunningham virus; mcMMAF = maleimidocaproyl monomethyl auristatin F; MMAE = monomethyl auristatin E; PBD = pyrrolobenzodiazepine; SN-38 = 7-ethyl-10 hydroxycamptothecin; SJS = Steven-Johnson syndrome; TEN = toxic epidermal necrolysis; Trop-2 = trophoblast cell surface antigen 2; VOD/SOS = hepatic veno-occlusive disease/sinusoidal obstruction syndrome

Despite their very promising design most of the currently-approved ADCs can cause severe and potentially life-threatening toxicities. Each component of the ADC, including the antibody, linker, and cytotoxic payload, may affect the extent of the ADC-induced toxicities (Table 5).^27^ Apart from myelosuppression, infections and TLS other important toxicities of these agents are interstitial lung disease (ILD)/pneumonitis, liver failure and skin toxicity (Table 5). Clinical symptoms of ILD/pneumonitis are generally nonspecific, including dyspnoea, cough and fever and can mimic infectious pneumonia. As pneumonitis can rapidly progress to a life-threatening condition, early consultation with the oncologist and the pulmonologist is recommended. The aim of management of ADC-related pneumonitis is to suppress inflammation and prevent the build-up of irreversible lung fibrosis. Therefore, the cornerstone of treatment of symptomatic (i.e. grade ≥ 2) ILD/pneumonitis is treatment with systemic corticosteroids (e.g. methylprednisolone 1−2 mg/kg/day) (Table 3). In very severe cases initial high-dose pulse corticosteroids (e.g. methylprednisolone 500−1000 mg/day for three days) is recommended. To prevent deterioration of ILD/pneumonitis systemic corticosteroids may be considered even in patients with asymptomatic (i.e. grade 1) ILD/pneumonitis who have only radiologic changes in their lung. Other immunosuppressive agents are recommended in refractory cases.^28^ The Steven-Johnson syndrome and toxic epidermal necrolysis are two forms of the same life-threatening skin disorder which cause rash, skin peeling, and sores of the mucous membranes. Treatment includes fluid replacement and nutrition, wound care, eye care, pain medication, medication to reduce inflammation of the eyes and mucous membranes, antibiotics to control infection systemic high-dose corticosteroids and IVIGs. Patients with these disorders usually require admission to the ICU.^29^

## Conclusions

The armamentarium of new systemic anticancer therapies is expanding rapidly. Clinical presentation of potentially fatal complications of these new therapies may be unpredictable and nonspecific and can mimic common medical conditions such as infections. Moreover, if not recognized and properly treated they can progress rapidly to life-threatening conditions. Patents with cancer who during their treatment develop acute illnesses may also present to GPs and EPs for initial workup. Therefore, it is very important that they are educated about side effects of new systemic anticancer therapies. Beside supportive care the mainstay of treatment of potentially severe toxicities of ICIs, CAR T-cells, BiTEs and sometimes of ADCs is systemic glucocorticoids and other immunosuppressive agents. In general, immunosuppressive therapy should start as soon as symptoms are mild-moderate. In patients with severe symptoms who require prompt immunosuppressive treatment concurrent empirical antimicrobial therapy may be started and continued until infectious cause of the acute illness is excluded. A multidisciplinary team involving a treating oncologist/haematologist and the corresponding organ-site specialist should be involved in the diagnosis and treatment of these treatment-related toxicities. In the changing landscape of oncology an establishment of cancer-specific urgent care centres might have an important role in the management of acutely ill patients with cancer.^30^
